# Biallelic *PDX1* (insulin promoter factor 1) mutations causing neonatal diabetes without exocrine pancreatic insufficiency

**DOI:** 10.1111/dme.12122

**Published:** 2013-02-28

**Authors:** E De Franco, C Shaw-Smith, S E Flanagan, E L Edghill, J Wolf, V Otte, F Ebinger, P Varthakavi, T Vasanthi, S Edvardsson, A T Hattersley, S Ellard

**Affiliations:** 1 Institute of Biomedical and Clinical Science, University of Exeter Medical SchoolExeter, UK; 2 Klinik für Kinder- und Jugendmedizin, St Vincenz-KrankenhausPaderborn, Germany; 3 TNM College and BYL Nair Charitable HospitalMumbai, India; 4 Kanchi Kamakoti CHILDS Trust Hospital and Childs Trust Medical Research Foundation (CTMRF)Chennai, India; 5 CentrallasarettetVaxjo, Sweden

## Abstract

**Aims:**

Recessive *PDX1* (*IPF1*) mutations are a rare cause of pancreatic agenesis, with three cases reported worldwide. A recent report described two cousins with a homozygous hypomorphic *PDX1* mutation causing permanent neonatal diabetes with subclinical exocrine insufficiency. The aim of our study was to investigate the possibility of hypomorphic *PDX1* mutations in a large cohort of patients with permanent neonatal diabetes and no reported pancreatic hypoplasia or exocrine insufficiency.

**Methods:**

*PDX1* was sequenced in 103 probands with isolated permanent neonatal diabetes in whom *ABCC8*, *KCNJ11* and *INS* mutations had been excluded.

**Results:**

Sequencing analysis identified biallelic *PDX1* mutations in three of the 103 probands with permanent neonatal diabetes (2.9%). One proband and his affected brother were compound heterozygotes for a frameshift and a novel missense mutation (p.A34fsX191; c.98dupC and p.P87L; c.260C>T). The other two probands were homozygous for novel *PDX1* missense mutations (p.A152G; c.455C>G and p.R176Q; c.527G>A). Both mutations affect highly conserved residues located within the homeobox domain. None of the four cases showed any evidence of exocrine pancreatic insufficiency, either clinically, or, where data were available, biochemically. In addition a heterozygous nonsense mutation (p.C18X; c.54C>A) was identified in a fourth case.

**Conclusions:**

This study demonstrates that recessive *PDX1* mutations are a rare but important cause of isolated permanent neonatal diabetes in patients without pancreatic hypoplasia/agenesis. Inclusion of the *PDX1* gene in mutation screening for permanent neonatal diabetes is recommended as a genetic diagnosis reveals the mode of inheritance, allows accurate estimation of recurrence risks and confirms the requirement for insulin treatment.

## Introduction

Permanent neonatal diabetes diagnosed before 6 months is a rare condition with a reported incidence of approximately 1:200 000 births in Caucasian populations 1. A genetic diagnosis is possible for ∼70% of cases 2, with the majority of patients harbouring mutation(s) in the potassium channel genes (*KCNJ11* or *ABCC8*) or in the *INS* gene 2.

Permanent neonatal diabetes attributable to pancreatic agenesis is very rare and, until recently, the causal gene was identified just in five cases, two of them caused by mutations in the *PTF1A* gene 3 and three with biallelic *PDX1* mutations 4–6. Recently, heterozygous mutations in the transcription factor *GATA6* were found to be the major cause of pancreatic agenesis in humans, with 15 cases reported 7.

PDX1 is a member of the homeodomain family of proteins necessary for pancreatic development. Mice with a homozygous null mutation of *Pdx1* lack a pancreas. *Pdx1*^+/–^ mice do not show any relevant phenotype at birth, but haploinsufficiency of *Pdx1* in β-cells causes impaired glucose tolerance in older mice (18 months) 8.

Biallelic mutations in *PDX1* are a known cause of pancreatic agenesis. The first homozygous *PDX1* mutation was reported in an infant with pancreatic agenesis 5, with a homozygous deletion of a single nucleotide, resulting in a premature termination codon (Pro63fsX60). The same mutation was later reported in a second case of pancreatic agenesis 6. A third case 4 was a compound heterozygote for two *PDX1* missense mutations (E164D, E178K) that were shown to decrease the protein half-life. Recently, Nicolino *et al*. 9 reported a family where two cousins with permanent neonatal diabetes and no clinical sign of pancreatic insufficiency harboured a homozygous missense mutation in *PDX1* (E178G). Biochemical studies revealed subclinical evidence of exocrine insufficiency in both infants. Functional studies showed that the E178G mutant protein had reduced transactivation activity, but normal localization, expression level and chromatin occupancy. They hypothesized that E178G is a hypomorphic mutation, causing the expression of a protein that still retains some residual activity, leading to a milder phenotype.

The finding that *PDX1* mutations can cause permanent neonatal diabetes in the absence of clinical features of exocrine pancreatic insufficiency prompted us to screen a cohort of 103 patients with isolated permanent neonatal diabetes to determine the contribution of biallelic hypomorphic *PDX1* mutations to the aetiology of this disease.

## Materials and methods

### Patient cohort

Mutation testing of *PDX1* was performed in 103 probands with isolated permanent neonatal diabetes diagnosed before 6 months (median age at diagnosis 13 weeks; interquartile range 1.5–24). The median birthweight was 2600 g (interquartile range 1800–3250). Seventeen out of 103 were born to consanguineous parents. Mutations in *ABCC8*, *KCNJ11* and *INS* had been excluded. We also excluded *EIF2AK3* mutations in patients born to consanguineous parents. None of the patients was reported to have pancreatic agenesis/hypoplasia or show clinical signs of exocrine insufficiency (defined as need for enzyme supplementation therapy). The relevant clinical information was provided by the referring clinicians. The study was conducted in accordance with the Declaration of Helsinki with informed parental consent given on behalf of children.

### Molecular genetic analysis

We screened the coding sequence and intron–exon boundaries of the *PDX1* gene (NM_000209.3) using Sanger sequencing at standard conditions and following manufacturers' protocols (primers available on request). For case IV we also sequenced the promoter region (3189 bp), the intron and the 3' untranslated region. Sequencing reactions were run on an ABI3730 capillary machine (Applied Biosystems, Warrington, UK) and analysed using Mutation Surveyor, v3.98 (SoftGenetics, State College, PA, USA). We used the bioinformatic tools SIFT and PolyPhen to predict the effect of novel variants on the PDX1 protein (protein reference NP 000200.1).

## Results

### Molecular genetic testing

Biallelic mutations in *PDX1* were found in three of the 103 probands with isolated permanent neonatal diabetes (2.9%) and a heterozygous nonsense mutation was identified in a fourth case.

**FIGURE 1:**
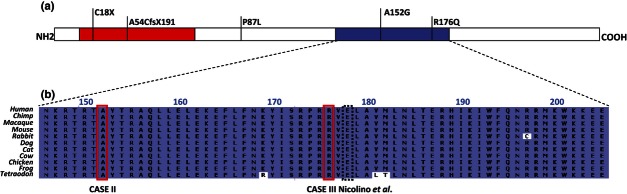
(a) Schematic representation of the PDX1 protein and location of the mutations identified in the four reported cases. The transactivation domain is highlighted in red. The region highlighted in blue is the homeobox domain of the PDX1 protein. (b) Amino acid conservation in the homeodomain. The red rectangles highlight the location of the two homeodomain mutations found in our cohort (cases II and III). The black dashed line indicates the position of the hypomorphic mutation reported by Nicolino *et al*. 9.

A male infant (I-1, Table 1) and his affected brother (I-2, Table 1) were compound heterozygotes for a frameshift and a novel missense mutation (p.A34CfsX191; c.98dupC and p.P87L; c.260C>T). The P87L mutation affects a residue located within the proline-rich region of the PDX1 protein (Fig. 1) and was inherited from the mother (see also Supporting Information, Fig. S1). Both the mother and her sister are homozygous for P87L (their parents are first cousins) and had gestational diabetes. The father, diagnosed with diabetes at 32 years of age, is heterozygous for the frameshift mutation and inherited the mutation from his unaffected mother.

**Table 1:** Clinical and molecular characteristics of five cases with neonatal diabetes and *PDX1* mutations

No.	Mutation	Protein	Birthweight	Age at diagnosis (days)	Pancreatic imaging result	Pancreatic imaging modality	Pancreatic exocrine: clinical	Pancreatic exocrine: biochemistry	Current insulin daily dose
I-1	c. 98dup/259C>T	A34CfsX191/P87L	2.5 kg/40 weeks	18	Normal pancreatic size	Ultrasound sonography	Asymptomatic	Faecal elastase 286 μg/g stool (Reference > 200 μg/g)	0.52 U/kg
I-2	c. 98dup/259C>T	A34CfsX191/P87L	2.76 kg/40 weeks	18	Normal pancreatic size	Ultrasound sonography	Asymptomatic	Faecal elastase 211 μg/g stool (Reference > 200 μg/g)	0.39 U/kg
II	c. 455C>G/455C>G	A152G/A152G	1.75 kg/40 weeks	2	Normal pancreatic size	Computed tomography abdomen	Asymptomatic	Not known	0.6 U/kg
III	c. 527G>A/527G>A	R176Q/R176Q	1.70 kg, gestational age not recorded	20	Normal pancreatic size	Ultrasound sonography	Asymptomatic	Not known	0.7 U/kg
IV	c. 54C>A/Normal	C18X/Normal	1.60 kg/37 weeks	8	Head of pancreas identified	Ultrasound sonography	Asymptomatic	Faecal chymotrypsin 0.36 mkat/kg (Reference > 0.32 mkat/l)	0.53 U/kg

A second proband (II, Table 1) harbours a homozygous mutation in exon 2 of *PDX1* (p.A152G; c.455C>G) and both unaffected parents are heterozygous. Another novel homozygous missense mutation (R176Q; c.527G>A) was found in a female infant (III, Table 1). Her unaffected father is heterozygous for the same missense mutation; DNA from the unaffected mother was not available for testing. Both the A152G and R176Q mutations affect highly conserved residues located in the homeodomain of PDX1 (Fig. 1) and were predicted to be deleterious by SIFT and PolyPhen-2.

Heterozygous *PDX1* base substitutions were found in four probands. One was a novel nonsense mutation (p.C18X; c.54C>A) (case IV, Table 1). Sequencing of the intron and regulatory regions (promoter and 3' untranslated region) of *PDX1* failed to identify a second mutation. Analysis of parental samples showed that the mutation was inherited from the unaffected father.

Two patients were heterozygous for synonymous variants (p.F100F; c.300C>T and p.P244P; c.732C>G) that are unlikely to be pathogenic. We also found a novel missense variant, p.P93R; c.278C>G that affects a residue within a highly variable region of the gene and is likely to be a rare polymorphism.

### Clinical characteristics of patients with *PDX1* mutations

The first family includes two siblings with permanent neonatal diabetes. Both the proband (I-1, Table 1) and his affected brother were diagnosed with diabetes at 18 days (blood glucose 20.9 mmol/l and 20.6 mmol/l, respectively). Both siblings are treated with insulin pumps. Ultrasound sonography showed a normal pancreas. There were no clinical features of exocrine pancreatic insufficiency and faecal elastase was within the normal range (Table 1).

Proband II and III were born to consanguineous parents (first cousins). Proband II was born at 40 weeks' gestation and developed diabetes at 2 days of life (44.4 mmol/l). The third patient (III, Table 1) was diagnosed with neonatal diabetes mellitus at 20 days (27 mmol/l). There was no clinical evidence of exocrine pancreatic insufficiency in either of these cases, but biochemical testing had not been performed as it was not indicated on clinical grounds.

Finally, we report a female infant in whom a single inactivating *PDX1* mutation was identified. The patient (IV, Table 1) was born to unrelated parents without diabetes. She was diagnosed with diabetes at 20 days and insulin treatment commenced. Ultrasound sonography was performed when she was 2 weeks old and showed that at least the head of the pancreas was present. Her faecal chymotrypsin level was normal.

## Discussion

Biallelic mutations in *PDX1* were identified in four patients from three families with isolated permanent neonatal diabetes. None were reported to have pancreatic agenesis or exocrine pancreatic insufficiency and, in the two cases where faecal elastase was measured, levels were normal.

The first patient and his affected brother are compound heterozygotes for a missense (P87L) and a frameshift mutation (A34CfsX191). Their mother was born to consanguineous parents and is homozygous for the P87L mutation. This mutation is likely to be hypomorphic as she did not develop diabetes until adulthood when she was diagnosed with gestational diabetes during her first pregnancy. The father is heterozygous for the frameshift mutation and any future offspring will be at 50% risk of permanent neonatal diabetes. The two other probands, both from consanguineous families, harbour homozygous missense mutations affecting highly conserved residues within the homeobox domain of the protein (respectively p.R176Q and p.A152G). The R176Q mutation affects a residue just two amino acids upstream of the mutation (E178G) reported by Nicolino *et al*. 9 (Fig. 1).

A fourth patient was found to be heterozygous for a novel *PDX1* nonsense mutation (C18X). The mutation is predicted to introduce a stop codon in the first exon of PDX1 and it is likely that the mutated transcript is subject to nonsense mediated decay. She inherited this mutation from her unaffected father but sequencing analysis of the intronic and regulatory regions of *PDX1* failed to identify a second, maternal mutation. This C18X mutation is predicted to cause neonatal diabetes in the homozygous, but not heterozygous state. Our results cannot distinguish between the alternative possibilities of an undetected maternal mutation (e.g. a translocation or inversion) or that the heterozygous mutation is coincidental to her phenotype, consistent with a previous report of heterozygous null *PDX1* mutations in population controls 10.

The first cases (three patients from two families) with biallelic *PDX1* mutations 4–6 had complete pancreatic agenesis. Subsequently, two cousins with permanent neonatal diabetes and subclinical exocrine insufficiency were reported 9. We report a further four cases with biallelic *PDX1* mutations without clinical evidence of exocrine pancreatic insufficiency. In the two cases tested, faecal elastase levels were normal. This suggests that the phenotype caused by biallelic *PDX1* mutations is correlated with the mutation type. Null mutations that abolish the protein activity cause pancreatic agenesis, while hypomorphic mutations partially affecting the protein functionality lead to permanent neonatal diabetes with or without exocrine insufficiency.

As expected, mutations in *PDX1* were more common in cases of isolated permanent neonatal diabetes born to consanguineous parents compared with outbred cases (2/17 vs. 1/86). Sequencing of *PDX1* is recommended in patients with isolated permanent neonatal diabetes, even in the absence of clinical or biochemical features of pancreatic exocrine insufficiency.

## Supporting Information

**Figure S1.** Partial pedigree of Family I. The arrow indicates the proband. Colors indicate phenotypic features: black = neonatal diabetes; shading grey = gestational diabetes; grey = adult-onset diabetes. N/N denotes no mutation identified.
